# Fibroblast growth factor 23: are we ready to use it in clinical practice?

**DOI:** 10.1007/s40620-020-00715-2

**Published:** 2020-03-04

**Authors:** Annet Bouma-de Krijger, Marc G. Vervloet

**Affiliations:** Department of Nephrology, Amsterdam Cardiovascular Science, Amsterdam University Medical Center, De Boelelaan 1117, 1081 HV Amsterdam, The Netherlands

**Keywords:** FGF23, Risk prediction, Cardiovascular disease, Clinical application

## Abstract

Patients with chronic kidney disease (CKD) have a greatly enhanced risk of cardiovascular morbidity and mortality. Over the past decade it has come clear that a disturbed calcium-phosphate metabolism, with Fibroblast Growth Factor-23 as a key hormone, is partly accountable for this enhanced risk. Numerous studies have been performed unravelling FGF23s actions and its association with clinical conditions. As FGF23 is strongly associated with adverse outcome it may be a promising biomarker for risk prediction or, even more important, targeting FGF23 may be a strategy to improve patient outcome. This review elaborates on the clinical usefulness of FGF23 measurement. Firstly it discusses the reliability of the FGF23 measurement. Secondly, it evaluates whether FGF23 measurement may lead to improved patient risk classification. Finally, and possibly most importantly, this review evaluates if lowering of FGF23 should be a target for therapy. For this, the review discusses the current evidence indicating that FGF23 may be in the causal pathway to cardiovascular pathology, provides an overview of strategies to lower FGF23 levels and discusses the current evidence concerning the benefit of lowering FGF23.

## Introduction

**Fig. 1:**
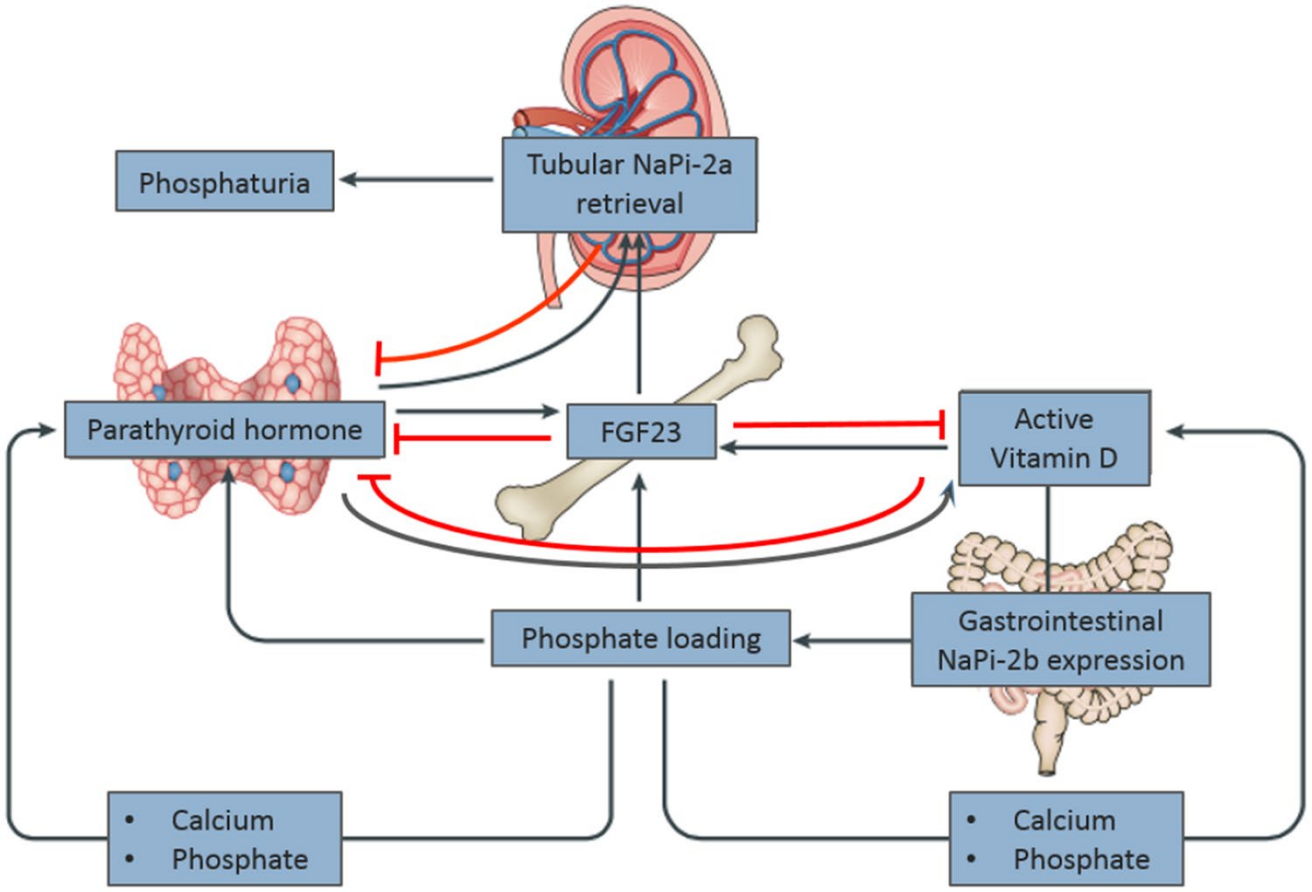
FGF23 physiology. Figure with permission adapted from Vervloet et al. Nature Reviews Nephrology 2017 (155) FGF23; Fibroblast growth factor 23, factor-23; Na-Pi-2a; Sodium-Phosphate co=transporter 2a

Chronic kidney disease (CKD) is a major health concern, given its high prevalence and associated cardiovascular morbidity and mortality, leading to a high rate of health care consumption [1]. This high burden of cardiovascular disease (CVD) is seen in CKD stage 3 and beyond. Although traditional risk factors, such as hypertension, diabetes and smoking contribute to the development of CVD in CKD, they cannot fully explain the high incidence of cardiovascular mortality in these patients [2, 3]. Disturbances in calcium-phosphate homeostasis are probably contributing to this high mortality risk [4]. A key hormone, in the regulation of calcium-phosphate homeostasis is the 32-kDa peptide Fibroblast Growth Factor-23 (FGF23). This hormone was discovered in the early 2000s in patients with autosomal dominant hypophosphataemic rickets (ADHR) [5]. This disease is characterized by hypophosphatemia and hyperphosphaturia resulting in growth retardation, bone deformities and rickets [6, 7]. FGF23 appeared to be the humoral factor to induce this excess renal phosphate loss. FGF23 is secreted by osteocytes in bone and is one of the three regulators of phosphate homeostasis, together with PTH and 1,25 dihydroxycholecalciferol (1,25(OH)_2_D_3_). The first two hormones both have phosphate lowering effects by decreasing tubular phosphate reabsorption by downregulation of the sodium dependent phosphate transporters (NaPi2a and NaPi2c) [8], but have opposite effects on vitamin D regulation, which is activated by PTH, but catabolized by FGF23 [9–12] (see Fig. 1). FGF23 acts on its main target organs, the kidney and parathyroid, by binding to the FGF23 receptors with α-Klotho as a co-receptor. This co-receptor is generally considered to be necessary to induce intracellular signal transduction, at least so for FGFR1 [13–15].

Nowadays FGF23 has gained wide attention in chronic kidney disease associated mineral bone disease (CKD-MBD) and appears to be a candidate as missing link between chronic kidney disease and cardiovascular morbidity and mortality. FGF23 levels increase during progression of CKD [16, 17]. Although this, initially physiological, adaptation is crucial for maintaining phosphate balance in early CKD, prolonged exposure and extreme concentrations in advanced CKD may have deleterious effects, in particular on the cardiovascular system [18–20]. Several observational studies showed an independent association between FGF23 levels and adverse outcome through all stages of CKD.

Over the last decade a legion of studies has been published on FGF23, unravelling its biology, physiological actions and its association with clinical conditions. Some epidemiological data and experimental studies suggest that FGF23 not only acts as regulator of parathyroid hormone (PTH), vitamin D, or phosphorus, but may actually be in the causal pathway to cardiovascular pathology. However, definite proof of causality is lacking, since many questions still remain. This review will focus on the clinical usefulness of FGF23 as a biomarker and its potential use as a target for therapy.

### Reliability of FGF23 measurement

Currently, FGF23 is rarely measured in routine clinical practice. There are four immunoassays commercially available for measurement of FGF23: Immutopics (1st and 2nd generation, San Clemente, USA), Kainos (Tokyo, Japan), Millipore (Billerica, USA) and DiaSorin (Saluggia, Italy). Most assays measure the intact 251 amino-acid protein (iFGF23) by simultaneous recognition of epitopes on the N- and C-terminal domains close to the proteolytic cleavage site. Additionally, Immutopics has an assay which measures both iFGF23 and the C-terminal fragment of FGF23 (cFGF23) by two antibodies against two epitopes within the C-terminal portion.

The four assays differ substantially as they are using different antibodies targeting different epitopes on the FGF23 protein. Besides different reported units [iFGF23 in picograms per milliliter (pg/ml) and cFGF23 in relative units (RU) per milliliter], absolute values between the assays vary substantially due to different calibration, and no harmonization has ever been conducted [21, 22].

If FGF23 would be used as a new biomarker certain issues need to be assessed. An ideal biomarker would be stable (no degradation ex vivo), show minimal diurnal variability and the analysis should be accurate, reproducible and affordable [23].

#### Stability of FGF23

There are several studies performed to assess the stability of FGF23, since intact FGF23 may be degraded by proteases or modified after blood withdrawal. First of all, iFGF23 is significantly more stable in plasma (EDTA) than in serum [24]. Even if samples after venepuncture are directly centrifuged and processed, there is the possibility of direct post-venepuncture instability of FGF23. The latter was investigated by Dirks et al. who found no differences between FGF23 concentrations in normal EDTA collecting tubes compared to tubes pre-coated with a protease-inhibitor, suggesting that no immediate protein proteolysis occurs after normal blood withdrawal [25].

However, when centrifugation is delayed a significant decrease of intact FGF23 concentrations was observed in several studies, both in healthy volunteers as well as in patients on dialysis, of 12% with the Immutopics assay, 7% with the Millipore assay and 5% with the Kainos assay (all p < 0.05) [25]. This confirmed two earlier studies that showed 23% reduction of FGF23 measured after a 8 h delay of centrifugation (compared with prompt centrifugation) using the second generation Immutopics assay [21]. Comparable reductions were found with the Millipore and Kainos assays [21, 26]. With the DiaSorin assay no such decrease in FGF23 concentrations was found [27].

Also, post-centrifugal stability of intact FGR 23 has been tested. No decrease of intact FGF23 concentrations are observed when in directly centrifuged samples delayed measurement of intact FGF23 was performed (after 8 h or more) with all the four currently used assays [25, 28, 29]. Besides, there are no indications of degradation of intact FGF23 after storage of processed samples at − 80 °C [25].

#### Biological variability

In healthy subjects, iFGF23 is subject to significant diurnal variation as iFGF23 concentrations peak in the early morning and fall during the day (mean decrease of 25%) [30, 31]. In contrast, cFGF23 concentrations show only a modest non-significant increase during the day [31]. The inequality of diurnal variation between iFGF23 and cFGF23 concentrations likely reflects the difference in clearance of the intact protein or its C-terminal fragment [32]. iFGF23 concentrations increase after phosphate intake with a delay of at least 12 h [31, 33]. Therefore it is preferable when measuring iFGF23 to use fasting samples or early morning samples as it is shown that iFGF23 concentrations in early morning samples are comparable to fasting samples [30]. In contrast, cFGF23 shows no significant postprandial changes in healthy individuals or patients with early CKD [31, 34, 35]. It is unlikely that a circadian rhythm is of clinical relevance in situations like advanced CKD, where FGF23 levels are extremely elevated.

#### Accuracy and reproducibility of methods for FGF23 measurement of different assays

In healthy adults, the 95% reference limits for plasma iFGF23 is 11.7–48.6 pg/ml and for cFGF23 21.6–91.0 RU/ml [30]. The intra- and inter-assay coefficient of variation are respectively, < 2.4% and < 4.7% for the second generation Immutopics cFGF23, < 9.7% and < 14% for the Kainos assay [36], < 2.9% and < 6.3% for the DiaSorin assay [28], and lastly, < 10% and < 8% for the Millipore assay. These latter variations are values provided by the manufacturer. The Millipore assay also reports to have a wider functional analytical range, this comes at the expense of poor sensitivity at low concentrations [21]. The first generation intact assay of Immutopics had an unacceptably high inter-assay coefficient of variation [36], that was substantially improved in the second generation assay to < 5% [37].

In patients on dialysis FGF23 concentrations become very high (frequently exceeding 100,000 RU/ml in prevalent patients). As the functional analytical range of the available assays is limited, large dilutions may be necessary to bring the concentration within this range. Another problem is that in patients on haemodialysis substantial intra-individual (week-to-week) variation in cFGF23 concentration has been reported [38, 39]. For all commercially available assays applies that they have not been validated for clinical use. Age- and renal function adapted reference ranges have not been established yet.

#### Using intact or c-terminal FGF23 assay and when?

The measurement of cFGF23 has, as earlier stated, the advantage of little diurnal variation and has more desirable variance characteristics with higher inter-individual than intra-individual variation. Besides, cFGF23 is more consistently associated with outcome as shown in the meta-analysis of Xiao et al. in which c-term FGF23 was associated with mortality in HD patients whereas iFGF23 measurement did not correlate to mortality [40]. Furthermore cFGF23 is a better predictor for identifying patients with declining renal function [41], atherosclerosis associated cardiovascular disease and heart failure [42]. Therefore, the cFGF23 assay may outperform the iFGF23 assay for clinical use, especially for the purpose of patients individuals risk assessment.

However, iFGF23 may better represent the biological effect of FGF23 [43], especially since it is reported that the c-terminal fragments might have counter-regulator effects to the biologically active full-length hormone [32]. A recent study on the effect of dietary phosphate restriction on FGF23 levels found a more pronounced effect on iFGF23, than on cFGF23 [44].

### FGF23 as a risk predictor

FGF23 is associated with progression of kidney failure and initiation of dialysis [41, 45–49]. This raises the question if FGF23 measurement might be a useful tool for risk prediction for progression of CKD and for other adverse outcome. Tangri et al. developed a model to predict progression of CKD that was validated in thirty-one cohorts, including 721,357 participants with CKD stages 3 to 5 (including age, gender, eGFR, albuminuria, serum calcium, serum phosphate, serum bicarbonate, and serum albumin) with a good performance of overall C-statistics (0.90; 95% CI 0.89–0.92). Several studies evaluated if FGF23 was able to improve this predictive value using specific statistics such as the area under the ROC curve and net reclassification index (NRI) statistics [50]. However, only one small study found that adding FGF23 to a base model improved the agreement of predicted and observed probability of renal function decline [51], all other studies found no significant improvement of risk prediction for decline in renal function or ESRD with the addition of FGF23 [49, 52–54].

However, for the prediction of all-cause mortality it has been reported that FGF23 might have added value in patients with CKD. [49, 53, 54] In a large study from the CRIC cohort by Edmonston et al. an improved prediction for all-cause mortality and hospital admission for heart failure was found when FGF23 concentration was added to the model, but the NRI did not reach statistical significance. For cardiovascular mortality no improved risk prediction was found [54]. Furthermore, in patients on hemodialysis, addition ofFGF23 did not improve risk prediction for mortality [55, 56].

Concerning cardiovascular events, FGF23 does not consistently improve prediction of novel events in hemodialysis patients [57]. Although one study showed that FGF23 improved prediction of fatal and non-fatal cardiovascular events in predialysis patients [49], another study showed small improvement of prediction only, yet without improved (NRI) classification. However, other studies showed no improvement at all [42, 54]. Interestingly the study by Emrich et al. found that when NT proBNP was added to the model the predictive value of FGF23 was largely eliminated and NT proBNP had a much stronger discriminating ability than FGF23 [42]. Overall, FGF23 only marginally improved the prediction for outcome. An overview of several risk predicting studies is provided in Table 1.

**Table 1:** Overview of several risk prediction studies using c-statistics, AUC and Net Reclassification Index (NRI)

References	Total no.	Follow-up, years	CKD stage	Risk predicting test	Improvement of risk prediction if FGF23 is added to the model			
					Renal failure	Cardiovascular events	Heart failure	mortality
Nowak et al. [52]	1032	5-12	Non-CKD, DMII	c-statistics AUC	No improvement for > 30% decline eGFR	–	–	–
Smith et al. [51]	171	2	3–4	c-statistics NRI	Improved for > 25% decline in eGFR (both c-statistics and NRI)	–	–	–
Levin et al. [53]	2544	1	3–4	c-statistics NRI	No improvement start RRT	–	–	Improved (both c-statistics and NRI)
Edmonston et al. [54]	3789	3	2–4	AUC NRI	No improvement for ESRD	No improvement (for atherosclerotic events)	Improved for hospital admission for heart failure (only with AUC, with NRI not significant)	Improved all-cause mortality (only with AUC, with NRI not significant) No improvement for CV mortality
Alderson et al. [49]	463	4	3–5	c-statistics NRI	Improved for start RRT (with c-statistics, NRI not significant)	Improved (with c-statistics, NRI not significant)	–	Improved (with c-statistics, NRI not significant)
Nakano et al. [57]	738	4	4–5, predialysis and follow-up into dialysis	NRI	–	Predialysis group: Prediction improved cardiovascular events (fatal and non-fatal) Dialysis group: no improvement		No improvement (both in predialysis and in dialysis group)
Yamashita et al. [56]	307	2	Haemodialysis	c-statistics	–	–	No improvement	–
Artunc et al. [55]	239	4	Haemodialysis	c-statistics	–	–	No improvement	–

Overview of several studies evaluating if FGF23 improves risk prediction using c-statistics, AUC and NRI, for risk prediction. C-statistic is equal to the area under a ROC curve and is a measure of goodness of fit for binary outcomes in a logistic regression model. NRI is a method to quantify if new marker provides clinically relevant improvements in prediction

*c-statistics* concordance statistic, *AUC* area under the curve, *NRI* net reclassification index, *FGF23* fibroblast growth factor 23, factor-23, *eGFR* estimated glomerular filtration rate, *iFGF23* intact FGF23*, RRT* renal replacement therapy, *ESRD* end stage renal disease

Future studies should evaluate whether multiple measurements of FGF23 may be advantageous compared to a single measurement for individual patients risk assessment in those with CKD, as it was shown that especially increasing FGF23 concentrations over time are associated with increased mortality [58, 59].

Apart from being a risk predictor, FGF23 might serve as an useful tool to identify patients to benefit from certain therapy. Udell et al. showed in their study among patients with stable ischemic heart disease that FGF23 was able to identify patients profiting from angiotensin-converting enzyme inhibitor therapy resulting in reduced cardiovascular death or incident heart failure [60].

### Indications of FGF23 toxicity from epidemiological studies

The question however arises if FGF23, besides being a potential risk predictor for adverse outcomes, might also have an instrumental role in the pathogenesis of complications. A great number of epidemiological studies sought an answer to this question.

#### Mortality

One of the first studies to report an association between FGF23 and mortality was the study by Gutierrez et al. [61] In this nested case control study among incident haemodialysis patients, a concentration dependent effect of FGF23 levels on mortality was observed. Even more interesting, this association became stronger after multiple adjustments, including adjustment for serum phosphate. This observation was confirmed in subsequent studies that followed, mainly in incident HD patients [62–65]. However, this finding is not consistent, as other studies found no association between FGF23 and mortality in patients on haemodialysis [66–70]. Overall, when 8 studies in patients on haemodialysis were pooled, a relative risk for the highest third of FGF23 versus the lowest third of FGF23 of 1.5 (95% CI 1.29–1.73) for all-cause mortality and of 1.42 (95% CI 0.96–2.39) for cardiovascular mortality was found the meta-analysis by Marthi et al. [71] Remarkably, the association of FGF23 with mortality is stronger in CKD patients not on dialysis despite much lower absolute levels of FGF23 [45, 46, 49, 53, 72–75]. Concerning the general population, although there are a few studies that found no association of FGF23 with all-cause mortality ([76, 77] most epidemiological studies (some consisting of great number of participants), report modest associations, even when adjusted for eGFR [75, 77–80].

#### Cardiovascular disease; myocardial infarction and stroke

In a post hoc analysis of the EVOLVE trial (vide infra) by Moe et al. among nearly three thousand patients on dialysis, FGF23 was statistically significantly associated with the incidence of myocardial infarction [81], an association also found in CKD [45, 75] and in the general population [82, 83]. However, for ischaemic stroke, no consistent association with FGF23 was found in in patient on dialysis [81], nor in the general population [75, 84]. Although some reports do suggest an association may exist with haemorrhagic stroke or thromboembolic stroke [82, 85, 86]. Concerning patients with pre-dialysis CKD, one cohort consisting of nearly four thousand patients found an association between FGF23 and a composite endpoint including myocardial infarction, stroke and peripheral vascular disease [87], an observation confirmed in other CKD cohorts and in the meta-analysis by Marthi et al. [45, 71, 75].

#### Left ventricular hypertrophy

There are epidemiological data linking FGF23 and left ventricular hypertrophy (LVH). The relatively small studies by Hsu et al. in 2009 and by Kirkpantur et al. in 2011 found an positive association between FGF23 and left ventricular mass in haemodialysis patients [66, 88]. However, in a sub analysis of the Evolve trial, among nearly three thousand haemodialysis patients, there was no association of FGF23 with heart failure [71, 81]. In CKD patients not on dialysis, the association with heart failure is more consistent. Although the study by Bouma -de Krijger et al. in in the Masterplan cohort found no association between FGF23 and congestive heart failure [73], other studies did report such an association [18, 75, 87, 89, 90]. Most epidemiological studies in the general population, one consisting of eleven thousand participants [79], also found an association between FGF23 and heart failure [79, 83, 91–93]. Combining several population studies [79, 83, 84, 91], the meta-analysis by Marthi et al. calculated a relative risk of FGF23 on heart failure of 1.24 (95% CI 1.29–1.69) for the highest versus the lowest tertile of FGF23 [71].

### Is there evidence that FGF23 can directly induce tissue pathology leading to organ damage?

Apart from its associations with clinical events in etiological driven epidemiological analyses, numerous experimental studies investigated potential mechanisms by which FGF23 might induce cardiovascular pathology.

#### FGF23 as a cause for left ventricular hypertrophy

Left ventricular hypertrophy (LVH) is an important contributor to cardiovascular morbidity in patients with CKD and LVH is associated with high FGF23 concentration [18]. Several studies explored the potential mechanisms by which FGF23 might induce LVH. For this, Faul et al. administered recombinant FGF23 (rFGF23) to isolated cardiomyocytes and to wild type and klotho deficient mice, where subsequently hypertrophic growth of the myocytes and cardiac hypertrophy in mice was observed, even so in the klotho knock-out animals [18]. FGF23 activated the FGF Receptor leading to calcineurin and nuclear factor of activated T cells (NFAT) signalling in cardiomyocytes. When a pan-FGF Receptor blocker was added to the CKD mice model, LVH was attenuated [18, 94]. Subsequent studies identified FGFR4 as the klotho-independent receptor for FGF23 on cardiomyocytes [95]. Specific blockade of FGFR4 by an antibody inhibited hypertrophy in the isolated cardiac myocytes and mice lacking the FGFR4 did not develop LVH in response to FGF23 [96], establishing FGFR4 as the receptor involved in FGF23-induced LVH [97]. However, these findings are not consistently reported, as in a transgenic mouse model of CKD, with high serum phosphate and FGF23, no signs of pathological cardiac remodelling were found [98].

Interestingly, LVH itself causes cardiac expression of FGF23. Matsui et al. developed two mice models of LVH. In both the transgenic and the pressure overload models, increased expression of FGF23 in the cardiomyocyte (inducing NFAT signaling) followed the development of LVH, while bone expression of FGF23 remained normal [99]. These findings were confirmed in an experimental animal models and in humans where, after myocardial infarction, expression of FGF23 in the heart is described [100, 101]. The upregulation of FGFR4 receptor, the culprit receptor for FGF23-induced cardiotoxicity, in the myocardium might further contribute to hypertrophy, which would imply a feedforward loop [102].

However, contradictory to the above findings, are the results of different mouse models of x-linked hypophosphatemia (XLH). Mice models of XLH have excess FGF23 production, yet those mice do not develop cardiac hypertrophy [103, 104]. This finding is confirmed in XLH patients, where no cardiac hypertrophy is observed [105]. There are several possible explanations for these contractionary findings. Unlike the situation in CKD, XLH is accompanied with low serum phosphate, normal blood pressure, serum calcium and renal function, and the absence of vascular calcification, and this very different phenotype may explain the discordancy. Also, in end stage CKD, FGF23 concentrations can reach values that are more than 1000 fold above normal, and as such much higher than in patients with XLH. Also, the different animal models of LVH had a variety of systemic alterations, such as high phosphate, uraemia or hypertension. It is possible that the deleterious effect of high FGF23 levels might result from a synergy between those abnormalities.

#### FGF23 and vascular calcification

Another mechanism contributing to the high burden of cardiovascular disease and mortality in CKD is arterial stiffness [106]. This can be worsened, among other causes, by vascular calcification or endothelial dysfunction. Arterial stiffness, regardless of its cause, increases pulse wave velocity, promotes the development of left ventricular hypertrophy, and can result in heart failure. An unresolved question is whether FGF23 can directly act on vascular cells to promote or inhibit matrix calcification. Two studies, by Scialla et al. and Lindberg et al., performed with vascular smooth muscle cells in vitro, showed no calcification when FGF23 was added [107, 108]. Also aortic rings or ex vivo mesenteric arteries of mice showed no calcification or changed vasoreactivity in response to FGF23 [107, 108]. On the other hand, Zhu et al. reported that addition of recombinant FGF23 had a protective effect on calcification in cultured murine SMCs [109]. In contrast, Jimbo et al. showed that FGF23 amplified Pi-induced calcification in cultured human vascular SMCs overexpressing α-Klotho [110]. Other studies focussed on the presence of α-klotho expression, necessary for FGFR1-mediated FGF23 action, in the vasculature. However, these results are also conflicting. Although some studies reported α-klotho expression and FGF23 signalling through FGFR1s receptor activation in the arterial wall [109–111], more compelling evidence refutes its presence [107, 112, 113].

#### FGF23 and endothelial dysfunction

As outlined, besides medial layer calcification, endothelial dysfunction can also contribute to arterial stiffness. Yilmaz et al. found a negative association between FGF23 and flow-mediated vasodilation (FMD) accompanied by increased concentrations of asymmetrical dimethyl arginine (ADMA), an endogenous competitive inhibitor of the vasodilator nitric oxide (NO) [114]. Since FGF23 and ADMA are both associated with progression of CKD, ADMA was added to a statistical model and was found to attenuate the effect of FGF23 on FMD. In two cohorts of CKD patients Tripepi et al. found a strong competitive interaction between FGF23 and ADMA suggesting that FGF23 is a modifier for ADMA levels, leading to dysregulation of the nitric oxide system associated with CKD progression [115].

Different experimental models have been used to explore this aspect of potential FGF23 toxicity. In an ex vivo model of isolated mice aortic rings, addition of recombinant FGF23 increased superoxide levels and reduced the bioavailability of nitric oxide in endothelial cells resulting in impaired relaxation [116]. When a pan FGF23 blocker was administered this effect was eliminated. Another experimental study suggested that the effect of FGF23 on endothelial cells is mediated by reactive oxygen species (ROS) that negatively influence arterial vasodilator capacity [117]. In that study mouse and human aortic rings (the latter obtained after aortic valve bypass surgery) and umbilical cord subjected to high concentrations of recombinant FGF23, recombinant soluble Klotho or phosphate in parallel showed increased ROS production. Addition of sKlotho attenuated the effect of FGF23 and Pi through increasing NO production, thereby protecting the vessel to some extend against the potentially noxious effects of high phosphate or FGF23 concentrations. In the study by Verkaik et al. resistance arteries from mice with renal failure and healthy mouse were studied ex vivo. They showed that pre-treatment with recombinant FGF23 impaired acetylcholine (Ach)-induced vasodilatation, which was restored after administration of FGF23 blocking antibodies regardless of the presence of renal failure.

Collectively, there is evidence that FGF23 induces arterial stiffness. This can be attributed to a large extent to impaired endothelial function, but unlikely to arterial calcification. This effect on endothelial layer mediated arterial stiffness would provide a rationale to target FGF23 as treatment goal. Obviously, before implementing such an approach, this needs clinical proof from prospective trials.

### Is FGF23 modifiable?

There are several potential strategies for reducing excess FGF23 levels or bioactivity, principally through dietary phosphate restriction or use of oral phosphate binders, by inhibiting FGFR signalling, by FGF23 blocking agents, and by the use of calcimimetics. In addition, in patients on dialysis, hemodiafiltration is able to reduce of FGF23 levels.

#### Dietary phosphate restriction to lower FGF23

Several studies evaluated if FGF23 could be reduced by the use of dietary phosphate restriction. Most studies in healthy individuals with normal kidney function reported a decline of intact FGF23 with dietary phosphate restriction and an increased iFGF23 after a phosphate-enriched diet. Studies in CKD found also a reduction of intact FGF23 with a phosphate restricted diet and increased FGF23 with dietary phosphate loading [118–120]. Interestingly, studies that measured C-terminal FGF23 did not report modification of cFGF23 with either phosphate loading or restriction, in healthy participants [31, 34, 35], as well as in patients with CKD [121, 122]. A possible explanation for this different findings for iFGF23 and cFGF23 was postulated by Smith et al. in their review on the different FGF23 assays [23]. They hypothesized that dietary phosphate loading might lead to enhanced FGF23 stability, thus a greater proportion of biologically active intact compared to the C-terminal peptide, in order to restore phosphate homeostasis. Of importance is that not only the absolute phosphate content in food counts, but also the source of phosphate matters, since bioavailability of phosphate is different between organic and inorganic phosphate. Phosphate as a food additive (for taste or conservation) is inorganic phosphate and is easily absorbed (bioavailability above 90%) compared to organic phosphate derived from vegetables such as in peas, nuts and cereals (absorption between 40 and 60%). The study by Moe et al. in CKD patients, elegantly showed that a vegetarian diet compared to a meat diet, despite both diets containing comparable amounts of phosphate, induced a decrease in iFGF23, whereas iFGF23 increased in the meat diet [123].

#### Lowering FGF23 by phosphate binders

Table 2 provides an overview of several different intervention trials using phosphate binders that also reported effects on FGF23 levels. When calcium-containing phosphate binders were used, all of six studies reported no decrease of FGF23 [124–129]. Block et al. even found in their placebo controlled trial among 148 patients with moderate CKD (eGFR 20–45 ml/min/1.73 m^2^), an increase of intact FGF23 with the use of calcium acetate as a phosphate binder [127]. In that study, in which patients were randomized to either placebo, lanthanum carbonate, calcium acetate or sevelamer carbonate, only the group of patients receiving sevelamer carbonate had a significant decline of intact FGF23 compared to placebo. Interestingly, these findings were assay-dependent as these results were not found when the c-terminal assay was used. Other prospective intervention studies that evaluated non-calcium based oral phosphate binders showed a similar pattern. Most studies in CKD patients measuring the intact FGF23 assay found a decrease of FGF23 with the use of either sevelamer [124–126, 128], or lanthanum carbonate [129–131] in CKD stage 2–5 patients. Ketteler and colleagues did report a 64% reduction of intact FGF23 using sucroferric oxyhydroxide (Velphoro) in patients on dialysis [132]. However, several other studies in predialysis CKD did not find a reduction of FGF23 levels with the use of sevelamer carbonate measuring intact FGF23 [133–136]. In addition, in several studies measuring c-terminal FGF23, no reduction was found after treatment with either lanthanum carbonate or sevelamer carbonate, sometimes even not so when combined with a low phosphate diet [121, 135, 137–140]. Only one of studies measuring c-terminal FGF23 found a reduction of FGF23 with dietary phosphate restriction [122]. However, the group with FGF23 reduction in this study had a higher baseline FGF23 concentration than the other groups making interpretation difficult. Another factor that might influence whether or not FGF23 reduction was achieved in the different studies, is the duration of phosphate binder use. Duration of therapy possibly should be 3-6 months or more since it is demonstrated that in kidney transplant patients high FGF23 levels may sustain for this period even when overt hypophosphatemia exists [141, 142], suggesting autonomous FGF23 production, which vanishes only after time. As outlined in Table 2, most studies were of relatively short duration.

**Table 2:** Overview of studies with phosphate binders with or without dietary phosphate restriction to lower FGF23

	Total no.	Study design	Duration of intervention	Age, years	Baseline characteristics			FGF23 assay	Intervention		Change in FGF23
					eGFR (ml/min)	Pi (mg/dl)	FGF23		Diet	Medication	
CKD											
Oliveira et al. [124]	40	Randomized trial, up titration med. every 2 weeks	6 weeks	50	35	3.5	97 pg/ml	Intact (Kainos)	Fixed 615 mg protein/day	Calcium acetate 1.32 g/day– > 2.64 g/day– > 5.3 g/day	No change
										Sevelamer HCT 1.6 g/day– > 3.2 g/day– > 6.4 g/day	Reduction
Isakova et al. [121]	16	2 × 2 factorial placebo controlled trial	2 weeks	62	40	3.2	158	c-term (Immutopics)	Pi diet 750 mg/day	Placebo	No change
									Pi diet 1500 mg/day	Lanthanum carbonate 3 g/day	No change
									Pi diet 750 mg/day	Lanthanum carbonate 3 g/day	No change
									Pi diet 1500 mg/day	Placebo	Increase
Gonzalez-Parra et al. [130]	18	Open label trial	4 weeks	70	42	3.5	212 RU/ml	c-term (Immutopics)	Fixed protein 0.8 mg/kg/day	Lanthanum carbonate 2250 mg/day	Reduction (22%)
Yilmaz et al. [125]	100	Randomized open label trial	8 weeks	45	24	7.7	40 pg/ml	Intact (Kainos)	NA	Sevelamer 1.6 g/day, up titrated to Pi < 5.5 mg/dl	Reduction (27%)
										Calcium acetate 3 g/day, up titrated to Pi < 5.5 mg/dl	No change
Bleskestad et al. [137]	21	Open label cross-over trial	2 × 2 weeks, 2 weeks wash-out in between	66	37	3.3	90/110 pg/ml	Intact (Kainos)	Ad Libitum diet	Alfacalcidol 0.25 μg/day– > Sevelamer 1.6 g/day	No change
										Sevelamer 1.6 g/day– > Alfacalcidol 0.25 μg/day	Increase (NS)
Vlassara et al. [126]	20	Open label cross-over trial	2 × 8 weeks, 1 week wash-out in between	61	38	4.0	95 μg/ml	NA (Genzyme diagnostics)	Ad Libitum diet, mean Pi intake 967 g/day	Sevelamer 4.8 g/day– > calcium carbonate 3.6 g/day	No change
										Calcium carbonate 3.6 g/day– > Sevelamer 4.8 g/day	Reduction in group with FGF23 baseline > 70 μg/ml
Block et al. [127]	148	Double blind RCT	9 months	68	32	4.2	120 pg/ml 227 RU/ml	Intact (Kainos) and c-term (Immutopics)	Ad Libitum diet	Calcium acetate av. 5.9 g/day	Increase
										Lanthanum carbonate av. 2.7 g/day	No change
										Sevelamer av. 6.3 g/day	Reduction of iFGF23, No change of cFGF23
										Placebo	No change
Isakova et al. [122]	39	2 × 2 factorial single blinded placebo controlled trial	12 weeks	55	38	3.6	129 RU/ml	c-term (Immutopics)	Ad Libitum diet	Placebo	No change
									Pi diet 900 mg/day	Lanthanum carbonate 3 g/day	No change
									Ad Libitum diet	Placebo	No change
									Pi diet 900 mg/day	Lanthanum carbonate 3 g/day	Reduction
Seifert et al. [135]	38	Double blind RCT	12 months	62/61	47/45	3.5/3.3	69/55 pg/ml	Intact (Kainos)	Ad Libitum	Placebo	No change
										Lanthanum carbonate 3 g/day	No change
Chue et al. [133]	109	Double blind RCT	36 weeks	55	50	3.2	69 pg/ml	Intact (Kainos)	Ad Libitum	Placebo	No change
										Sevelamer 1.6 g/each meal	No change, reduction only when adherence to medication was ≥ 80%
Spatz et al. [138]	40	Prospective open cohort study	12 weeks	70	21	4.8	602 RU/ml	c-term (Immutopics)	Verbal instruction for low Pi diet	Sevelamer 2.4 g/day up titrated on serum Pi	No change
Liabeuf et al. [134]	78	Double blind RCT	12 weeks	63	27	3.8	157 RU/ml, 72 pg/ml	c-term and intact (both Immutopics)	Ad Libitum	Sevelamer fixed dose 4.8 g/day	No change (both iFGF23 and cFGF23)
										Placebo	No change
Bouma-de Krijger et al. [140]	24	Prospective open cohort study	8 weeks	52	44	3.5	167 RU/ml	c-term (Immutopics)	Ad Libitum	Sevelamer fixed dose 4.8 g/day	No change
Ix et al. [136]	205	Double blind RCT (COMBINE trial)	12 months	69	32	3.7	99 pg/ml	Intact (Kainos)	Verbal instruction for low Pi diet	Nicotamide 225 mg/day +placebo	No change
										Nicotamide 225 mg/day + lanthanum carbonate 3 g/day	No change
										Placebo + lanthanum carbonate 3 g/day	No change
										Placebo + placebo	No change
Dialysis											
Koiwa et al. [128]	46	Open label randomized trial	4 weeks	57	Dialysis	5.9	9000 ng/l	Intact (manufacturer NA)	NA	Sevelamer 3 g/day + calcium carbonate 3 g/day	Reduction
										Calcium carbonate 3 g/day	No change
Brandenburg et al. [139]	75	Open label cohort study	8 weeks	65	Dialysis	6.4	7244 RU/ml	c-term (Immutopics)	NA	Sevelamer titration on top of usual Pi-binder medication to serum Pi < 5.5 mg/dl	No change
Chang et al. [129]	25	Open label randomized trial	8 weeks	56/61	Dialysis	6.8/6.5	8678/8565 pg/ml	Intact (Kainos)	Pi diet 600-800 mg/day	Lanthanum carbonate up titrated to serum Pi	Reduction
										Calcium carbonate up titrated to serum Pi	No change
Zhang et al. [131]	92	Open label randomized trial	12 months	48	Dialysis	8.0/7.7	348/328 pg/ml	Intact (Biochamp China)	NA	Lanthanum carbonate up titrated to serum Pi < 5.5 mg/dl	Reduction
										Calcium carbonate up titrated to serum Pi < 5.5 mg/dl	No change
Ketteler et al. [132]	1059	Multi centre open label phase 3 study	24 weeks and a subset of no. 549 up to 1 year	56	Dialysis	7.4	39600 pg/ml	Intact (Immutopics)	NA	Sucroferric hydroxide 1–3 g/day	Reduction at 24 and at 52 weeks
										Sevelamer 2.4–14.4 g/day	Reduction at 24 and at 52 weeks
											Pooled analysis 64% reduction at 52 weeks

Overview of studies with phosphate binders with or without dietary phosphate restriction to lower FGF23

*FGF23* fibroblast growth factor 23, factor-23, *eGFR* estimated glomerular filtration rate, *iFGF23* intact FGF23, *c-term* carboxy terminal fragment of FGF23, *Pi* serum phosphate, *NA* not available

#### FGF23 antibodies and FGF23 receptor blockers

Burosumab is an FDA approved monoclonal antibody targeting FGF23 and was developed for the treatment of XLH. In FGF23-mediated hypophosphataemic disorders it improves hypophosphatemia and bone abnormalities in children [143]. However, there are no data on its use in CKD and its use might be even induce harm by disturbing the adaptive response of FGF23, as was shown in an experimental model of CKD [144]. In that animal study, FGF23 antibodies resulted in decrease of FGF23 and ameliorated uremic hyperparathyroidism. However, urinary phosphate excretion decreased and hyperphosphatemia developed, promoting vascular calcification and increased mortality. The same observations were found in an experimental study with a pan-FGFR inhibitor [145], showing that FGF23 remains necessary to maintain serum Pi levels within range, at least in non-dialysis dependent CKD. A more promising strategy might be to specifically inhibit FGFR4 signalling to prevent the development of LVH associated with high FGF23 levels [96]. However, this was only tested in an animal model and there are currently no data available to suggest a clinical benefit for patients with CKD.

#### Calcimimetics

Several studies showed that FGF23 concentrations can be reduced by the use of cinacalcet or etelcalcetide. In a randomized trial by Wetmore et al. it was demonstrated that patients assigned to cinacalcet compared to low dose calcitriol had a decrease of serum FGF23 concentrations [146]. However, it was not clear if this was caused by the relatively low dose of active vitamin D in the cinacalcet treatment arm, considering that active vitamin D being a strong stimulator of FGF23. The study by Koizumi et al. found that the decrease of FGF23 during cinacalcet treatment was independent of active vitamin D [147]. Also data from the Evolve trial showed that cinacalcet is a potent suppressor of FGF23 [81]. The newer intravenously used calcimimetic etelcalcetide appears to be an even more potent suppressor of FGF23 compared to cinacalcet [148]. However, the mechanism by which cinacalcet or etelcalcetide suppresses FGF23 is unclear. Both a direct effect on the calcium sensing receptor on osteocytes or a decline of the calcium-phosphate product may have accomplished this effect.

#### Haemodiafiltration (HDF)

Previous studies have shown that FGF23 with its 32 kDa middle molecular size can be cleared by HDF and not by low-flux HD [149]. Several studies have shown a percent reduction of serum FGF23 within a single HDF session of around 50% (±  25%), with FGF23 detected in spent dialysate samples [150–152]. The study by Bouma et al. showed a sustained decline FGF23 over time, of greater size with higher convection volume [70].

### Is it useful to measure serial FGF23 concentrations?

Whether change of FGF23 better reflects risks and possibly impacts on outcomes is an open question. There are some studies that do shed some light in this issue. Probably the best of these is the study by Isakova on the CRIC cohort, consisting of 1135 patients with a mean eGFR of 46.3 (± 14.7) ml/min per 1.73 m^2^. Although, the majority of patients from this cohort had stable FGF23 concentrations during the 5 years of follow-up, patients with rapidly increasing FGF23 concentrations had an exceptionally high mortality risk [58]. Also, in a sub analysis of the CONTRAST study in prevalent haemodialysis patients, increasing levels of FGF23 were associated with increased mortality [70]. Both studies found, in different populations, that increasing FGF23 is disadvantageous. However, the study by Jovanovich in over 900 dialysis patients showed that, although over 24 month stable low FGF23 concentration was associated with a favourable outcome compared to stable high FGF23 concentrations, the group with high FGF23 in which FGF23 further increased over time, had no further increased risk for all-cause mortality [59]. More importantly, this study also showed that in patient with high baseline, and subsequently decreasing FGF23 concentrations had no improved risk for mortality. Likewise, in the sub analysis of the CONTRAST study, a decrease in FGF23 was not associated with improved mortality compared to a stable FGF23 concentration. A secondary analysis of the EVOLVE trial [153] analysed the impact of cinacalcet-induced reductions in FGF23. This analysis, different from the studies discussed above, did suggest that decreasing FGF23 might be beneficial [81]. Here, a more than 30% reduction of FGF23 in 20 weeks among participants allocated to cinacalcet, was associated with a reduced risk on the composite outcome of cardiovascular mortality, sudden cardiac death and heart failure. Remarkably, in the placebo treated group there were also (yet fewer) patients with more than 30% FGF23 reduction, but in those patients no association with reduced risk on outcome was found. This suggests that not the decline of FGF23 itself, but the way it was achieved, determined the more favourable outcome. Since the main outcome of the EVOLVE trial demonstrated no improvement of the primary combined outcome in cinacalcet treated patients, FGF23 reduction probably just identified patients who might benefit more from cinacalcet treatment [153]. Since FGF23 has been implicated with the development of LVH, Seifert et al. studied CKD patients with an increase of LVH over 12 months. This worsening of LVH was not associated with an increase of FGF23 [154]. Moreover, Chue et al. identified in their study, among CKD patients treated with sevelamer, a subgroup with a decrease of FGF23, which was however not accompanied with a change in arterial stiffness, left ventricular mass or cardiac function [133]. Therefore, there is currently no compelling evidence that FGF23 reduction leads to improved outcome and this leaves the role of FGF23 in the pathway to adverse outcome still under debate.

### FGF23 are we ready to use it in clinical practice? (conclusion)

In conclusion, FGF23 is a promising biomarker in CKD. Although the different FGF23 assays should be harmonized and assay specific reference intervals should be established, FGF23 measurement has shown to be consistently associated for adverse outcome in different populations. Furthermore, FGF23 measurement was shown, and validated, to predict mortality in CKD. However, FGF23 has limited value in predicting progression of renal failure in CKD or in risk prediction in patients on dialysis. Future studies should evaluate the predictive validity of repeated FGF23 testing. Increasing FGF23 concentrations over time, both in CKD and dialysis patients are associated with dismal outcomes.

Considering the fact that there is currently no, or very limited, evidence that FGF23 reduction leads to improved outcome, it is preliminary to use FGF23 concentrations as target for therapy in everyday clinical practice, despite the ability of dietary phosphate restriction and phosphate binders to lower FGF23 concentrations in CKD. For patients on haemodialysis both calcimimetics, non-calcium containing phosphate binders and HDF are effective modes to lower FGF23. The availability of interventions that lower FGF23 sets the stage for clinical trials that target FGF23 (and not phosphate concentrations) and have clinical events as primary endpoint.
